# The Enigma of Interspecific Plasmodesmata: Insight From Parasitic Plants

**DOI:** 10.3389/fpls.2021.641924

**Published:** 2021-04-01

**Authors:** Karsten Fischer, Lena Anna-Maria Lachner, Stian Olsen, Maria Mulisch, Kirsten Krause

**Affiliations:** ^1^Department of Arctic and Marine Biology, UiT The Arctic University of Norway, Tromsø, Norway; ^2^Central Microscopy at the Biology Center, Christian-Albrechts-University, Kiel, Germany

**Keywords:** *Cuscuta*, feeding hyphae, haustorium, interspecific plasmodesmata, parasitic plants, secondary plasmodesmata, symplasm

## Abstract

Parasitic plants live in intimate physical connection with other plants serving as their hosts. These host plants provide the inorganic and organic compounds that the parasites need for their propagation. The uptake of the macromolecular compounds happens through symplasmic connections in the form of plasmodesmata. In contrast to regular plasmodesmata, which connect genetically identical cells of an individual plant, the plasmodesmata that connect the cells of host and parasite join separate individuals belonging to different species and are therefore termed “interspecific”. The existence of such interspecific plasmodesmata was deduced either indirectly using molecular approaches or observed directly by ultrastructural analyses. Most of this evidence concerns shoot parasitic *Cuscuta* species and root parasitic Orobanchaceae, which can both infect a large range of phylogenetically distant hosts. The existence of an interspecific chimeric symplast is both striking and unique and, with exceptions being observed in closely related grafted plants, exist only in these parasitic relationships. Considering the recent technical advances and upcoming tools for analyzing parasitic plants, interspecific plasmodesmata in parasite/host connections are a promising system for studying secondary plasmodesmata. For open questions like how their formation is induced, how their positioning is controlled and if they are initiated by one or both bordering cells simultaneously, the parasite/host interface with two adjacent distinguishable genetic systems provides valuable advantages. We summarize here what is known about interspecific plasmodesmata between parasitic plants and their hosts and discuss the potential of the intriguing parasite/host system for deepening our insight into plasmodesmatal structure, function, and development.

## Introduction

Symplasmic domains are operational units which are formed by joining the protoplasts of cells by way of plasmodesmata (PD) that form complex structures across the plant cell walls (Ehlers and Kollmann, 2001) or by sieve pores that originate from PD (Kalmbach and Helariutta, 2019) but are limited to the sieve elements of the phloem. Based on when and where they originate, two different types of PD are distinguished: primary PD originate during cell division, while secondary PD are formed across already existing cell walls. Despite their different origin, no structural differences can be discerned between them (Burch-Smith et al., 2011). Studies of secondary PD have, therefore, focused on non-division walls, which are of ontogenetically different origin and contain exclusively secondary PD (Ehlers and Kollmann, 2001). While this is a convenient system for structural analyses, a challenge that remains is to delineate the chain of molecular events that regulates secondary PD formation. To this end, the study of PD formed between genetically different plants promises a possibility to distinguish the molecular steps in each of the two cells that contribute to their establishment. Such interspecific PD (iPD) are by definition secondary as they are inserted in principle into existing cell walls of two unrelated individuals. Such a situation occurs either in graft unions (Kollmann and Glockmann, 1985, 1991) or at the interface between parasitic plant haustoria and the invaded tissue of their hosts (Dörr, 1969; Lee, 2009). While grafting is limited to closely related species of a few angiosperm families, some parasitic plants infect a wide range of distantly related host plant species encompassing both monocots and dicots (Westwood et al., 2010).

Parasitic plants, by definition, procure part or all of their nutrients from autotrophic plants, which serve as their hosts. Having initially evolved from fully photoautotrophic ancestors, they now occupy a narrow and specialized but apparently lucrative niche – given that the evolution of parasitic lineages has taken place many times independently within the angiosperms (Nickrent, 2020). The specialized lifestyle has led to various adaptations of which the invention of an infection organ, termed haustorium, was the primary key to their success (Yoshida et al., 2016). The term haustorium refers to the tissue of the parasite that develops endophytically within the infected host plant and is a morphological trait that is common to all parasitic plants (Smith et al., 2013). Unlike their fungal counterparts, parasitic plant haustoria are complex multicellular organs. With them, parasites can invade either the shoots (e.g., dodders, mistletoes) or the roots (e.g., broomrapes) of their hosts and withdraw either only water and inorganic nutrients through xylem connections (hemiparasites) or inorganic plus organic compounds via connections to host xylem, phloem, and parenchyma cells (holoparasites).

**Figure 1:**
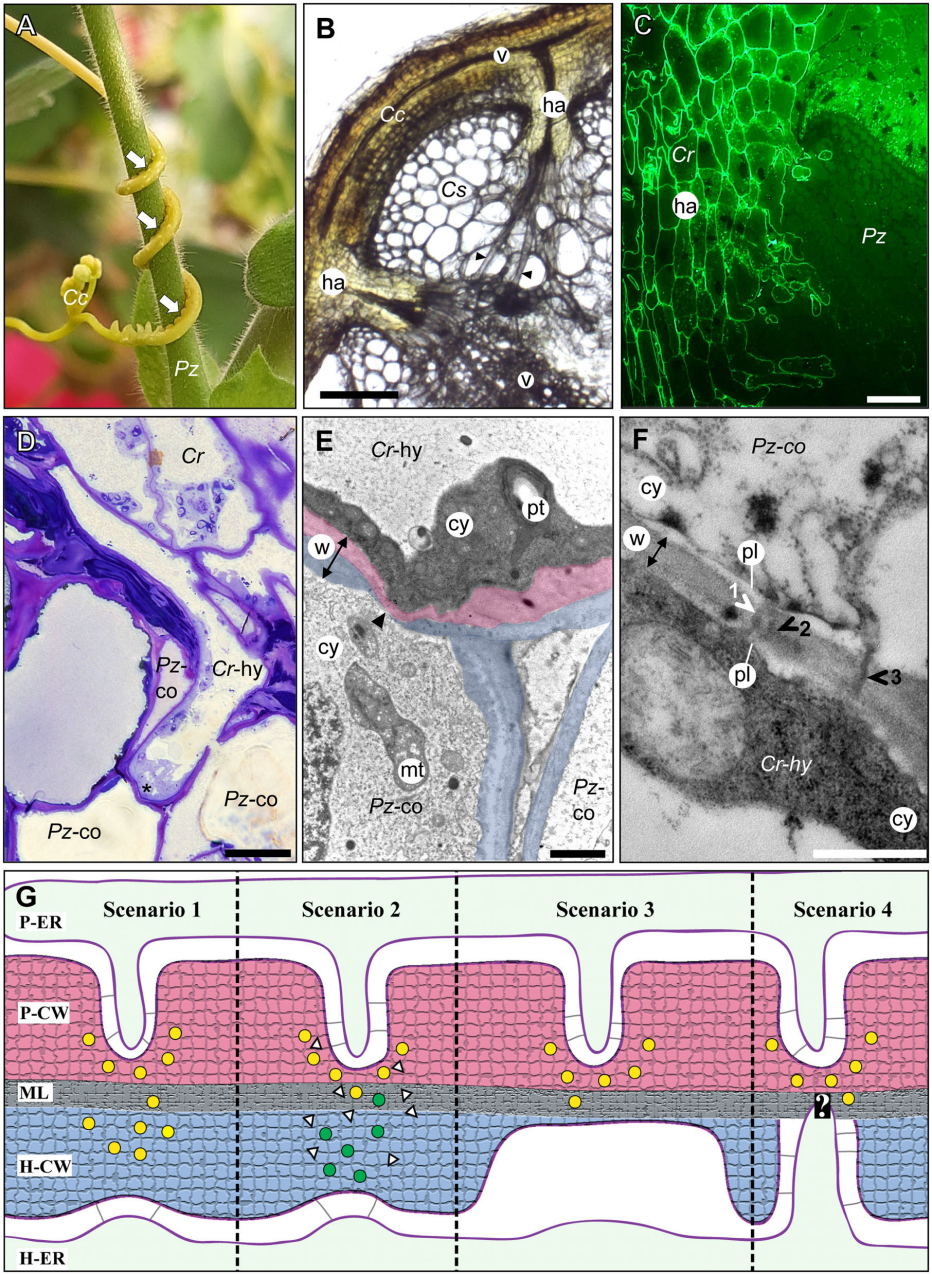
The host/parasite feeding interface. **(A)** The yellow vine *C. campestris* (*Cc*) twines around its host *Pelargonium zonale* (Pz) making infection sites (arrows) where parasitic haustoria penetrate the host tissue. **(B)** Light micrograph of a transverse vibratome section of *C. campestris* (*Cc*) infecting *Cucumis sativus* (*Cs*) revealing the endophytic haustoria (ha) with their protruding hyphae (black arrowheads) that connect both plants' vascular elements (v). Scale bar: 300 μm. **(C)** Fluorograph of an immunolabeled microtome cross section of a parasite/host boundary. A monoclonal antibody (JIM8) against arabinogalactan proteins selectively labels *C. reflexa* (*Cr*) cell walls but not cell walls of the host *P. zonale* (*Pz*) and enables the precise identification of the haustorium (ha) interface. Scale bar: 100 μm. **(D)** Light micrograph of a toluidine blue-stained section showing a hypha (*Cr*-hy) of the parasite *C. reflexa* (*Cr*). The hypha has grown through one host cortex cell (*Pz*-co) and is in the process of penetrating another (site marked with an asterisk, *). Scale bar: 20 μm. **(E)** Electron micrograph of the hypha (*Cr*-hy) shown in **(D)** penetrating a host cortex cell (co). The thinned or ruptured host cell wall is marked with an arrowhead. Parasite (*Cr*) and host (*Pz*) cell walls are highlighted with red and blue shading, respectively. The cell wall (w, with double-sided arrow indicating its width), cytoplasm (cy), host cell mitochondrion (mt), and parasite plastid (pt) are labeled. Scale bar: 2 μm. **(F)** Electron micrograph of a cell wall (w) between a *C. reflexa* hypha (*Cr*-hy) and a penetrated *P. zonale* cortex cell (*Pz*-co). Three plasmodesmata (1, 2, and 3) are marked with arrowheads that are colored either white where they connect to both cells' plasmalemma (pl) and black where they appear to cross the wall only partially. PD 2 appears to be branched, while the others are seemingly unbranched PD. Scale bar: 0.5 μm. cy = cytoplasm. **(G)** Schematic illustration of four hypothetical scenarios (Scenarios 1–4) how PD formation at the parasite/host interface could be coordinated. Cell walls are shaded with red (parasite) and blue color (host) like in **(E)**. Cell wall enzymes secreted to thin/loosen the cell walls are represented by yellow (from parasite) or green (from host) dots. In Scenario 1, the parasite-secreted enzymes are moving across the middle lamellae (ML) to act on the host cell wall (H-CW). In Scenario 2, unknown signals (white triangles) from the parasite induce the release of host cell wall enzymes (green dots) to autodecompose their cell walls locally. In Scenario 3, the parasite cell wall enzymes are secreted in a location where the host wall is already thin [see situation at hyphal tips in **(E)**]. In Scenario 4, the parasite cell wall enzymes are secreted in a location where a pre-infection host PD is present. The white question mark indicates that this scenario is the most speculative because it assumes that the parasite is able to locate the host PD. The association of parasite ER (P-ER) and host ER (H-ER) with their respective plasma membranes is indicated by gray lines. The methods used to generate the microscopy images are described in the Supplementary Materials file.

One parasitic plant genus that has been classified as a noxious weed in many countries is *Cuscuta* (dodder) (Figure 1A). *Cuscuta* species are destructive shoot parasites due to their broad host spectrum that includes annual plants and perennial shrubs and trees from most orders within the angiosperm lineage (Vogel et al., 2018). The endophytic haustorium of *Cuscuta* species protrudes from the center of a suction cup-like ring, the adhesive disk, which anchors the parasite to the host surface (Vaughn, 2002; Lee, 2007). At an early stage of infection, the haustorium penetrates the host plant surface by applying mechanical pressure and releasing cell wall degrading enzymes that weaken the host tissue cohesion (Vaughn, 2003; Johnsen et al., 2015). Following this initial invasion, the haustorium expands and grows through the cortex and often the sclerenchymal ring in search of the vascular tissue of the host. At the final stages of the infection, elongated cells (so-called searching and feeding hyphae) emerge from the tips and flanks of a haustorium (Figure 1B). The active feeding stage usually only lasts for a limited time, and the process of nutrient acquisition is taken over by younger haustoria as the parasite grows and finds new hosts.

The haustorial hyphae form physical and physiological bridges between host and parasite (Figures 1C–E) and facilitate the nutrient and water transfer. The hyphae appear to recognize which host cell type they approach, and they differentiate into a matching cell type (Vaughn, 2006). Thus, xylem vessels of the host, which are comprised of tube-like dead cells are intercepted by xylem-like (xylic) hyphae that re-direct water and minerals to the parasite (Christensen et al., 2003). On the other hand, amino acids, sugars, and other organic molecules in the phloem sap are channeled to the parasite through phloic hyphae that surround the host sieve elements (Dörr, 1972; Hibberd and Jeschke, 2001; Birschwilks et al., 2006; Vaughn, 2006). Hyphae connecting to parenchymal host cells show fewer morphological changes but are characterized by an electron-dense and organelle rich cytoplasm (Dörr, 1969). Chimeric cell walls and symplasmic connections between the different hyphae and the host tissue provide cohesion between the partners and it is tempting to also assume that they ensure the efficiency in nutrient uptake that the parasite depends on.

## Evidence for Symplasmic Connections Between Holoparasitic Plants and Their Hosts

Although investigations of cytoplasmic contacts between parasitic plant haustoria and the infected host tissue are not exceptionally abundant, indirect and direct evidence for iPD at the host/parasite interface has accumulated over the past half century (Table 1).

**Table 1:** Summary of studies investigating cell-to-cell connections between parasitic plants and their hosts.

		**Species**	**Host cell type**		**Interspecific symplasmic connection**	**Experimental method**						**References (chronologically sorted for each category)**
	**Parasite**	**Host**	**Parenchyma**	**Phloem**		**EM**	**IL^a^**	**FP^b^**	**FS^c^**	**RT^d^**	**V^e^**	
**Ultrastructural studies**	*O. cumana*	*Helianthus annuus*		●	SP	●						Krupp et al., 2019
	*C. japonica*	*Impatiens balsaminea*		●	PD, SP	●						Lee, 2009
	*C. reflexa*	*Arabidopsis thaliana*	●		PD	●						Birschwilks et al., 2007
	*C. platyloba*	*Arabidopsis thaliana*	●		PD	●						Birschwilks et al., 2007
	*C. odorata*	*Arabidopsis thaliana*	●		PD	●						Birschwilks et al., 2007
	*C. reflexa*	*Vicia faba*	●		PD	●						Birschwilks et al., 2006
	*C. platyloba*	*Nicotiana tabacum*	●		PD	●						Birschwilks et al., 2006
	*C. odorata*	*Nicotiana tabacum*	●		PD	●						Birschwilks et al., 2006
	*C. pentagona*	*Impatiens balsaminea*	●		PD	●	●					Vaughn, 2006
	*C. pentagona*	*Impatiens sultanii*	●		PD	●	●					Vaughn, 2003
	*O. crenata*	*Vicia narbonensis L*.	●	●	PD, SP	●						Dörr and Kollmann, 1995
	*C. odorata*	*Pelargonium zonale*	●		PD	●						Dörr, 1969
**Macromolecular transport**	*P. ramosa*	*Brassica napus*		●	SP				●			Peron et al., 2016
	*P. aegyptiaca*	*Solanum lycopersicum*		●	SP		●	●	●			Ekawa and Aoki, 2017
	*P. aegyptiaca*	*Solanum lycopersicum*		●	SP			●				Aly et al., 2011
	*C. reflexa*	*Arabidopsis thaliana*		●	SP			●	●	●		Birschwilks et al., 2007
	*C. odorata*	*Arabidopsis thaliana*		●	SP			●	●	●		Birschwilks et al., 2007
	*C. platyloba*	*Arabidopsis thaliana*		●	SP			●	●	●		Birschwilks et al., 2007
	*C. reflexa*	*Vicia faba*		●	SP			●	●	●	●	Birschwilks et al., 2006
	*C. odorata*	*Nicotiana tabacum*		●	SP			●	●	●	●	Birschwilks et al., 2006
	*C. platyloba*	*Nicotiana tabacum*		●	SP			●	●	●	●	Birschwilks et al., 2006
	*C. reflexa*	*Nicotiana tabacum*		●	SP			●	●			Haupt et al., 2001

*Ultrastructural studies provided direct evidence for the presence of interspecific symplasmic connections, while molecular studies provided indirect evidence for their existence based on macromolecular transport analysis. The main experimental approaches in each study (EM, electron microscopy; IL, immunolabeling; FP, fluorescent protein transport; FS, fluorescent stain; RT, radioactive tracer labeling; V, virus movement) are indicated. PD, plasmodesmata; SP, Sieve pores*.

a *Callose antibody*.

b *AtSUC2-GFP, Tobacco mosaic virus movement protein-GFP, ER-targeted GFP*.

c *5,6-carboxyfluorescin diacetate (CFDA) for transport studies or aniline blue for callose staining*.

d *14C or 3H*.

e *potato virus Y isolate N*.

### Physiological and Molecular Evidence From the Genus *Cuscuta*

In contrast to mineral nutrients and small organic compounds that in plants take both apoplastic and symplastic transport routes (Offler et al., 2003; Zhang and Turgeon, 2018), macromolecules (proteins or nucleic acids) require symplasmic connections, either in the form of PD between neighboring cells or through sieve pores or sieve plates between sieve elements (Kalmbach and Helariutta, 2019). A nice demonstration of macromolecular transport between host plants and *Cuscuta* and, with it, unequivocal proof for a continuous and efficient connection between parasite and host vascular bundles was provided using the green fluorescent protein (GFP) (Haupt et al., 2001; Birschwilks et al., 2007). That the exchange of proteins in fact occurs at a large scale was recently shown through a proteomics approach (Liu et al., 2020). Several 100 host proteins were identified in *C. australis* growing on *A. thaliana* or soybean and, surprisingly, hundreds of *Cuscuta* proteins were found in the two host plants, indicating a massive bidirectional protein movement. Furthermore, mRNAs were found to move from host to parasite (Roney et al., 2007; David-Schwartz et al., 2008; LeBlanc et al., 2013) and this happens at a genomic scale involving transcripts of thousands of genes (Kim et al., 2014). MicroRNAs are also shuttled from the parasite to the host to target host gene expression (Shahid et al., 2018; Johnson and Axtell, 2019). Last but not least, plant viruses have for 75 years been known to move between *Cuscuta* and its host plants (Bennett, 1944; Mikona and Jelkmann, 2010), a transmission also depending on PD. Collectively, these data point to a massive flow of substances in both directions that cannot be explained by apoplastic translocation alone but necessitates open symplasmic connections between *Cuscuta* and its hosts. There are to date no molecular studies that explain how this massive flow could be regulated or to what degree it is selective.

### Ultrastructural Evidence From the Genera *Cuscuta* and *Orobanche*

Despite the molecular data discussed above, there is only limited ultrastructural evidence for symplasmic connections between parasite and host vascular tissues (Table 1). In the root parasitic genus *Orobanche* a connection between parasite and host via sieve elements has been convincingly shown for *O. crenata* connecting to *Vicia narbonensis* (Dörr and Kollmann, 1995) and for *O. cumana* parasitizing *Helianthus annuus* (Krupp et al., 2019). In both cases, interspecific sieve plates were observed. For the shoot parasite *Cuscuta*, in contrast, compelling evidence for sieve plates at the parasite/host border is still lacking. Claims regarding sieve pores between phloic hyphae of *Cuscuta japonica* and sieve elements of *Impatiens* (Lee, 2009) were not supported by visual evidence and have not been confirmed when the same host was infected with *Cuscuta pentagona* (Vaughn, 2003, 2006). However, several accounts of plasmodesmata between host parenchyma cells and *Cuscuta* searching hyphae have been published (Table 1). Such investigations revealing iPD have used five different *Cuscuta* species infecting an even larger range of different hosts from genera like *Pelargonium, Vicia, Impatiens, Nicotiana* or *Arabidopsis* (Dörr and Kollmann, 1995; Vaughn, 2003, 2006; Birschwilks et al., 2006, 2007). The reports differ with respect to the abundance of iPD and it was proposed that they may be relatively short-lived and present only in hyphae from the younger parts of the haustorium while they seem to degenerate later (Dörr, 1969; Vaughn, 2003). Both authors provided very detailed descriptions of the versatile iPD structures with unbranched and complex branched forms with visible desmotubules occurring side by side. Vaughn (2003) also described collars and fibrillar spokes radiating out from the desmotubule, suggesting that their ultrastructure could be very similar to that of PD that connect cells from the same organism. Later stages were observed to contain occlusions or appear to fuse and form hairpin loops running back to the same cell, but it should be kept in mind that the reports show 2-dimensional snapshots of a complex system and both the spatial and temporal dimensions have not been investigated. Therefore, caution should be exercised when interpreting findings of incomplete iPD (see Figure 1F and literature cited in Table 1). This notion, together with the still unexplained sustained transport activities, calls for higher temporal resolution of haustorial development and additional modern technologies in future studies of the host/parasite connections.

## Establishment of Interspecific Secondary PD

iPD are a special case of secondary PD as they span the cells of different individuals, species and even higher order phylogenetic lineages. So far, very little is known about this type of PD.

### Control of Secondary PD Formation

Some evidence suggests that PD do not develop from one side only, but that they are formed in a coordinated process by the two opposing cells (Kollmann and Glockmann, 1991; Ehlers and Kollmann, 2001). The process is believed to start with a local thinning of the cell wall on both sides followed by the trapping of ER cisternae which develop into plasmodesmal desmotubules, the fusion of the two plasma membranes and finally the reconstruction of the cell wall (Ehlers and Kollmann, 2001; Burch-Smith et al., 2011). If both cells contribute to the formation of complete secondary PD, some kind of communication across the cell borders is needed. Potential scenarios how this could happen are depicted in Figure 1G. It should be noted that these are hypothetical alternatives and experimental insight regarding the regulation of secondary PD formation and the molecules involved in signaling is lacking. Whether PD initiation happens unilaterally by one cell in a given tissue or starts simultaneously in two neighboring cells, is also unresolved. While in the parasite/host system it is presumably the parasite that initiates PD formation as this connection appears to be vital for the parasite's survival, it is likewise still unclear how and how much the host contributes (Figure 1G).

### Cell Wall Degradation and Rebuilding

Cell wall breakdown and rebuilding are thought to be important steps of secondary PD formation (Ehlers and Kollmann, 2001; Burch-Smith et al., 2011). In the case of intraspecific PD the two parts of the common cell wall and the enzymatic machinery for the cell wall remodeling are in principle identical. The cell walls of the host and parasite, on the other hand, do differ to some degree (Johnsen et al., 2015) (Figure 1C) and accordingly the enzymes involved in remodeling the cell walls are also expected to differ. It is well-known that during invasion of the host the parasite secretes a cocktail of enzymes which degrade the cell walls of the host but not their own (Nagar et al., 1984; Losner-Goshen et al., 1998; Olsen et al., 2016). Host cell walls abutting haustorial cells were observed to have a lower degree of pectin esterification than walls that were not in contact with the haustorium (Johnsen et al., 2015). Young hyphae were also often found to be surrounded by host cell walls that were stretched extremely thin [Figures 1D,E and Vaughn (2003)]. This provided evidence for extensive deconstruction and loosening of the host cell walls at the site of contact, but it remains speculative whether this thinning is mediated by host or parasite enzymes (see Figure 1G, scenarios 1 and 2). With cell wall degradation products being discussed as potential signaling molecules for cell wall integrity (Ferrari et al., 2013), the parasite's enzymes could tentatively contribute to the coordination of PD formation between parasite and host by inducing host enzyme secretion in corresponding places (Figure 1G, scenario 2). Alternatively, similar signals may help the parasite identify regions with thinned host walls and induce PD formation in these regions (Figure 1G, scenarios 3 and 4).

### iPD in Graft Unions

Besides parasitic/host interfaces, graft unions are sites where interspecific symplasmic connections can potentially be formed. Already Jeffree and Yeoman (1983) observed cell wall thinning and formation of plasmodesmata in opposing cells of autografted tomato (*Solanum lycopersicum*) plants. More pertinent, Kollmann and colleagues were able to show iPD in heterografts between different species (Kollmann et al., 1985) and different orders (Kollmann and Glockmann, 1985, 1991). Anatomically, both full and partial unbranched connections as well as complex branched PD were described, thus resembling closely what has been found at the parasite/host interface. Cell wall thinning seemed to precede the PD formation in the described cases. Using serial sections, Kollmann and Glockmann (1985) could show that apparently incomplete “half” iPD were in fact continuous structures connecting both adjacent cells. However, this seems to depend on the cell types that align with each other and “half PD” that end at the middle lamella were found where the alignment was not perfect (Kollmann et al., 1985). Diffusion through graft interface iPD was demonstrated using fluorescein in grafts between different Prunus species (Pina et al., 2009), demonstrating the functionality of these structures in transport.

## Parasitic Plants As Tools for the Analysis of Secondary PD

Secondary iPD at the host/parasite border are an excellent system to overcome limitations of current PD research for several reasons. First, the symplasmically connected partners have different genotypes, and form many more different combinations than grafting currently offers. This facilitates the identification of the origin of the genes and proteins involved in the establishment of secondary PD, which could finally answer the question whether the PD are initiated uni- or bilaterally. Moreover, the searching and feeding hyphae of the parasite can be faithfully distinguished based on their characteristic ultrastructure (Dörr, 1969, 1972; Vaughn, 2003, 2006) or on unique epitopes in their cell walls (Vaughn, 2003; Johnsen et al., 2015) (Figure 1C). Thus, the border between parasite and host tissues and thereby the location of heterospecific cell walls can be precisely mapped. The parasite/host system therefore allows detailed analyses of the roles that each of the two symplasmically connected cells have in this process. In contrast, in successful grafts the two partners are often very closely related, making such differentiation more challenging, if not impossible.

Second, quite many parasitic plants, including the well-researched *Orobanche* and *Cuscuta*, can infect many different hosts (Yoshida et al., 2016; Shimizu and Aoki, 2019). Their host range includes popular model plants like *A. thaliana*, tobacco or tomato and thus offers the opportunity to harness all molecular genetic tools developed for those. Among them, a plethora of transgenic and mutant lines (overexpressing lines, knock-out lines, introgression lines) are available and have already been used to dissect parasite/host interactions (Hegenauer et al., 2016; Krause et al., 2018). Classical transgenic technology, RNA interference (Mansoor et al., 2006) and genome editing technologies like CRISPR-Cas9 (Doudna and Charpentier, 2014) are available for many compatible hosts. Furthermore, whole genome sequences and large-scale transcriptomic datasets are available for hundreds if not soon thousands of plants (Wong et al., 2020). On the parasite side, the first genome sequences have been published for *Cuscuta* (Sun et al., 2018; Vogel et al., 2018). Although transgenic parasitic plants cannot yet be produced efficiently, recent progress gives reason to believe that genetic manipulation of these parasites will soon be a standard (Lachner et al., 2020).

With the development of new methodology for tracing symplasmic transport via non-invasive approaches and suitable biotracers, the origin and fate of enzymes and structural components and maybe even of signaling molecules might in the future be traceable or even manipulated unilaterally using interspecific interfaces in parasites, but also in grafts.

## What Can We Learn About PD Using the Parasite/Host System?

The basic structure of primary and secondary PD is very similar (Brunkard and Zambryski, 2017; Sager and Lee, 2018). The ER membranes and the plasma membranes of the two cells are fused and span the PD to provide a symplasmic connection. However, it is unclear whether the fusion resembles well-described membrane fusion processes, e.g., those between vesicles and the plasma membrane, or whether it is completely different. In the parasite/host system the protein composition and most likely also the lipid composition of the membranes of the two cells are sufficiently different to be of benefit for more detailed analyses of the fusion process.

Proteins also contribute to the structure of PD (Sager and Lee, 2018). Although in the last decades many proteins localized in PD have been identified (Han et al., 2019), their physiological and molecular functions are mostly unknown. It is not even known if the proteins are contributed by one or both cells. The different genotypes of the host and parasite cells provide an optimal instrument to answer such developmental questions.

Transport through PD changes during the course of plant development and in response to stress, and is therefore tightly controlled through the size exclusion limit (SEL) or pore size, or by closure of the PD (Brunkard and Zambryski, 2017). Although some factors regulating transport through PD such as light, the circadian clock (Brunkard and Zambryski, 2019) or sugars (Brunkard et al., 2020) have been identified recently, there is limited knowledge about PD regulation at the physiological and molecular level. Only a few molecules regulating SEL have been characterized. Among them are virus movement proteins which increase SEL to allow movement of viruses in a process called gating. In the parasite/host system similar processes are assumed to take place and it is tempting to speculate that this is achieved by “gating molecules” produced by the parasite to prevent closing of the PD by the host. Indeed, it has been proposed that the control of the common host/parasite symplast is the key characteristic of compatible interactions (Cheval and Faulkner, 2017), a claim that could be tested by investigating the iPD.

## Conclusion

iPD established between parasitic plants and their hosts offer a unique perspective on symplasmic domains and secondary PD in general. They promise to be an advantageous system to address and answer open questions regarding their formation and regulation. In particular, the respective contribution of neighboring cells can be analyzed and discriminated. Considering that adequate molecular tools for the parasites are only now beginning to emerge, we will hopefully see many new pieces of valuable information generated in this highly contemporary field in the future.

## Data Availability Statement

The original contributions presented in the study are included in the article/Supplementary Material, further inquiries can be directed to the corresponding author.

## Author Contributions

All authors listed have made a substantial, direct and intellectual contribution to the work, and approved it for publication.

## Conflict of Interest

The authors declare that the research was conducted in the absence of any commercial or financial relationships that could be construed as a potential conflict of interest.
