# Supplementary material for: The Enigma of Interspecific Plasmodesmata: Insight From Parasitic Plants
Source: Front Plant Sci. 2021 Apr 1;12:641924. doi: 10.3389/fpls.2021.641924 (PMC8049502; doi:10.3389/fpls.2021.641924)
Supplement: Supplementary file 1 [file Data_Sheet_1.PDF]

Supplementary Material:

MATERIAL AND METHODS

Plant material

The *Cuscuta* species *C. reflexa* and *C. campestris* were maintained in a greenhouse on *Pelargonium zonale* as host. New *P. zonale* were infected with parasite shoot tips around 20 cm length from a growing culture as described by Krause et al. (2018). *Cucumis sativus* plants were infected with *C. campestris* seedlings from germinated seeds. Visual inspection was used to select mature sites for microscopical analyses.

Imaging of fresh infection sites

Vibratome sections of infection sites without prior fixation were prepared as described in detail by Olsen and Krause (2019) using a Leica VT1000 E vibrating blade microtome (vibratome). Light micrographs were taken with an Axio Observer Z1 (Zeiss).

Anatomical imaging and immunolabelling of fixed infection sites

Procedures for semi-thin sectioning, immunolabelling and toluidine blue staining are described by Krause et al. (2018). Briefly, infection sites were trimmed into approximately 1-2 mm thick pieces and were subjected to fixation in 1% glutaraldehyde and 1% formaldehyde in PEM buffer (pH 6.9). After washing in standard phosphate buffered saline solution (1x PBS), dehydration in an ethanol series was performed and the samples were embedded in gelatine capsules containing London Resin (LR) White resin. Semi-thin sections (1 µm) were collected on coated glass slides and were stained with Toluidine Blue O for anatomical observations. Immunolabeling was performed with the monoclonal antibody JIM8 (PlantProbes, Leeds, UK) using a described labelling protocol (Krause et al. 2018). Samples were then mounted in anti-fade mounting medium and observed in a Leica DMRBE microscope equipped with a color camera.

Transmission electron microscopical imaging of fixed infection sites

For ultrastructural analysis, infection sites were trimmed as described above, fixed in 2.5% glutaraldehyde and 1% formaldehyde in Na-cacodylate buffer (pH 7.2) and postfixed in 1% (w/v) OsO_4. After dehydration in a graded series of ethanol, embedding was done in LR white resin. After further trimming of the blocks to the host/parasite interface, ultrathin sections were collected on pioloform-coated Cu-grids, stained with lead citrate and saturated uranyl acetate in water and observed in a Philips CM10 transmission electron microscope (Grabowski et al., 2008).

References

Grabowski, E., Miao, Y., Mulisch, M., and Krupinska, K. (2008). Single-stranded DNA-binding protein Whirly1 in barley leaves is located in plastids and the nucleus of the same cell. *Plant Physiol.* 147, 1800-1804. doi: 10.1104/pp.108.122796

Krause, K., Johnsen, H. R., Pielach, A., Lund, L., Fischer, K., and Rose, J. K. C. (2018). Identification of tomato introgression lines with enhanced susceptibility or resistance to infection by parasitic giant dodder (*Cuscuta reflexa*). *Physiol. Plant.* 162, 205-218. doi: 10.1111/ppl.12660

Olsen, S., and Krause, K. (2019). A rapid preparation procedure for laser microdissection-mediated harvest of plant tissues for gene expression analysis. *Plant Methods* 15, 88. doi: 10.1186/s13007-019-0471-3
